# Transcriptomic Analysis Reveals the Molecular Mechanisms of Prolactin in Regulating Porcine Follicular Development

**DOI:** 10.3390/genes16070774

**Published:** 2025-06-30

**Authors:** Yubin You, Beibei Han, Qiang He, Li Li, Shouquan Zhang, Hengxi Wei

**Affiliations:** State Key Laboratory of Swine and Poultry Breeding Industry, National Engineering Research Center for Breeding Swine Industry, Guangdong Provincial Key Laboratory of Agro-Animal Genomics and Molecular Breeding, College of Animal Science, South China Agricultural University, Guangzhou 510642, China; youyubin1118@163.com (Y.Y.); hy426234234@163.com (B.H.); heqiangshi158@163.com (Q.H.); ili007@scau.edu.cn (L.L.); sqzhang@scau.edu.cn (S.Z.)

**Keywords:** prolactin, granulosa cell, follicular development, proliferation, angiogenesis

## Abstract

Background: Prolactin (PRL) is a key reproductive hormone that regulates follicular development through endocrine and paracrine mechanisms. However, its specific role in porcine follicular development remains unclear. Methods: In the in vivo experiments, follicular fluid and tissue cells were obtained from small (1–2 mm), medium (3–4 mm), and large (5–6 mm) porcine follicles. PRL levels in follicular fluid were measured by ELISA. The expression levels of genes and proteins related to follicular development were assessed using quantitative real-time PCR (RT-qPCR) and Western blotting (WB). In the in vitro experiments, CCK-8, RT-qPCR, and WB were used to detect the effects of different concentrations (0, 30, and 300 ng/mL) of recombinant porcine prolactin (prPRL) on granulosa cell (GC) proliferation, steroid hormone synthesis, and angiogenesis, and transcriptome sequencing was performed. Results: The PRL concentration was significantly higher in large follicles compared to small and medium follicles. During follicular development, expression levels of PRL, PRL receptor (PRLR), proteolytic enzymes (CTSD, MMP2, MMP14, and BMP-1), and angiogenic factors (VEGFA and FGF-2) increased and then decreased. Moreover, prPRL promoted GC proliferation, increased the expression of PCNA and cyclin D1, upregulated steroidogenesis-related genes *CYP11A1* and *3β-HSD*, and significantly enhanced the expression of key angiogenic factors VEGFA and FGF-2. RNA-seq analysis identified 226 differentially expressed genes (DEGs), which were mainly enriched in signaling pathways such as the Hippo, JAK/STAT, and Rap1 pathways. Conclusions: PRL may regulate porcine follicle development by affecting cell proliferation and angiogenesis in GCs through the Hippo, JAK/STAT and Rap1 signaling pathways.

## 1. Introduction

Prolactin (PRL) is predominantly synthesized by the anterior pituitary gland, which initiates downstream signaling pathways through its interaction with the prolactin receptor (PRLR), causing various physiological and biochemical responses in target cells [1]. Granulosa cells (GCs) are the major somatic cells of follicles and play essential roles in follicular development and oocyte maturation. The expression of PRLR in granulosa cells indicates that prolactin directly influences the function of these cells [2]. In previous studies, 4 ng/mL of PRL enhanced the proliferation of sheep GCs, whereas the apoptosis of sheep GCs was promoted by using 500 ng/mL of PRL [3,4]. PRL may be involved in the proliferation and apoptosis of ovarian GCs in a concentration-dependent way [5]. The proliferation and apoptosis of GCs play crucial roles in follicular development and determine follicular growth, maturation, and entry into the ovulatory stage. Follicular development and estrus in sows are, in part, regulated by weaning; therefore, studying the regulatory role and mechanism of PRL in follicular development in pigs has scientific significance and production value.

PRL has a broad spectrum of biological activity involving nearly all major physiological processes, including reproduction, development and growth, immunomodulation, proliferation and differentiation of cells, endocrinology, and metabolic processes [6,7,8,9]. Of particular interest is the ability of PRL to modulate the steroid secretion and angiogenic factors in GCs [10,11]. Steroid hormone synthesis and angiogenesis are central factors in the regulation of follicular development. Pro-angiogenic factors include vascular endothelial growth factor (VEGF), basic fibroblast growth factor (FGF-2), platelet-derived growth factor (PDGF), and angiopoietin (ANGPT), which promote endothelial cell proliferation and migration [1]. Steroid hormones, including estrogens, progestogens, and androgens, play different roles at different stages of follicular development, such as promoting processes of follicular growth, maturation, and ovulation [12], and angiogenesis is essential for follicular growth and maturation and its transition to the corpus luteum [13]. Steroid hormones and angiogenesis also have synergistic effects during follicular development; steroid hormones induce the expression of angiogenic factors and promote angiogenesis, thereby improving follicular development and ovulation. During follicular development, angiogenesis ensures effective hormone delivery and response, which helps maintain a healthy environment for follicular development [14]. Caspase D (CTSD), bone morphogenetic protein-1 (BMP-1), and matrix metalloproteinases (MMPs) are essential enzymes that play a crucial role in the degradation of the extracellular matrix and tissue remodeling as well as angiogenesis [15]. Studies have found that PRL is cleaved by CTSD, BMP-1, and MMPs to produce vasoinhibin (Vi), which inhibits the growth of capillary endothelial cells. The production of Vi is closely linked to the expression and activity of associated proteases that regulate PRL levels in the hypothalamus, pituitary, and target tissues. The relative levels of PRL and Vi play crucial roles in angiogenesis [16]. Follicular development involves a complex series of coordinated events. In addition to steroidogenesis, angiogenesis, and cell proliferation, the immune response plays a key role in many physiological reproduction events [17]. During follicular growth, inflammatory cytokines regulate the secretion of steroids required for reproduction, ovulation, and other functions [18,19].

Although the effects of PRL on GCs have been partially investigated, the mechanisms and potential roles regarding the action of PRL in porcine ovaries, especially in GCs, remain unclear. Therefore, this work explored the expression patterns of PRL, PRLR, and some key genes associated with granulosa cell proliferation, steroid hormone synthesis, and angiogenesis during follicular development using both in vitro and in vivo investigations, as well as investigating the potential regulatory signaling pathways at the transcriptional level through transcriptome sequencing. This approach facilitates a thorough understanding of the various regulatory mechanisms of PRL in porcine follicle formation and establishes a scientific foundation for enhancing reproductive efficiency in the pig breeding business.

## 2. Materials and Methods

### 2.1. Experimental Reagents

Recombinant porcine PRL (prPRL) was prepared as previously described [20]. Follicle-stimulating hormone (FSH; Ningbo Second Hormone Factory, Ningbo, China) and androstenedione (Aladdin, Shanghai, China) were used. Unless otherwise stated, all other reagents were obtained from Gibco (Thermo Fisher Scientific, Waltham, MA, USA).

### 2.2. Experimental Design

**Figure 1:**
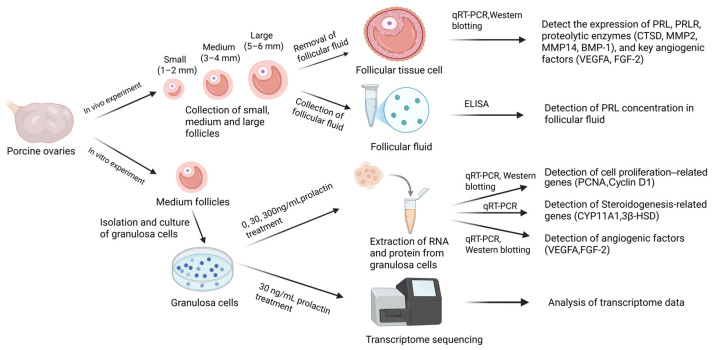
Experimental flowchart (created in Biorender).

To investigate the regulatory mechanisms of PRL in follicular development, we carried out both in vitro and in vivo studies. In the in vivo experiments, the PRL concentration in the follicular fluid of small, medium, and large follicles was determined. In addition, the basal expression of PRL, PRLR, proteolytic enzymes, and angiogenic genes that are important for follicular development were assessed in isolated follicles of varying sizes. In the in vitro experiments, granulosa cells were extracted from healthy medium-sized follicles, cultured, and subjected to varying doses of prPRL (0, 30, and 300 ng/mL). The effects of PRL on follicular development were examined by evaluating cell proliferation, steroid hormone synthesis, angiogenesis-related gene expression, and transcription results. The experimental design is illustrated in Figure 1.

### 2.3. Collection of Ovaries

Porcine ovaries were harvested from healthy gilts (Landrace, about 6 months old, weighing about 120 kg) at a commercial slaughterhouse (Kong Wang Ji, Guangzhou, China). Fresh ovaries were rinsed twice with prewarmed saline and then placed in saline containing a streptomycin/penicillin mixture (1%). The ovaries were rinsed thrice with phosphate-buffered saline (PBS) at 37 °C and transported to the laboratory within 2 h.

### 2.4. Collection of Follicular Fluid and Follicular Tissue Cells

The follicles were excised from the ovaries using a surgical blade, ophthalmic scissors and forceps under a stereomicroscope. Based on the morphological criteria, follicles were categorized as atretic or healthy, with atretic follicles characterized by opaque or “milky” follicular fluid [11]. Healthy follicles were then classified into small (1–2 mm), medium (3–4 mm), and large (5–6 mm) groups according to their diameters, as measured using vernier calipers. The follicles were punctured repeatedly with a 1 mL syringe to release the follicular fluid and centrifuged at 3000 rpm. The supernatant follicular fluid was collected and used for ELISA, and the lower layer of precipitated follicular cells was stored at −80 °C.

### 2.5. ELISA

Following the manufacturer’s guidelines, PRL concentrations in the follicular fluid were measured using an ELISA kit (Ruixing Biotech, Guangzhou, China). Standard solutions (50 μL) with different concentrations were added to the corresponding standard wells, while 50 μL of sample solutions were added to each well. Then, 100 μL of antibody solutions were dispensed into each well. After adding 50 μL of the substrate, the 96-well plate was rinsed five times with wash solution and incubated for 15 min at 37 °C. Subsequently, 50 μL of the stopping solutions were added, and each well’s optical density (OD) was assessed at 450 nm utilizing a microplate reader. A standard curve was constructed by plotting the concentrations of the standard solutions (x-axis) versus their corresponding OD values (y-axis), allowing for the calculation of the sample concentrations using the curve equation.

### 2.6. Culture of Granulosa Cells

GCs were isolated and cultured as previously described [21]. Briefly, GCs were extracted from follicles with a syringe and subsequently centrifuged at 1100 rpm for 5 min. The supernatant was removed, and the cells were rinsed twice with PBS containing a streptomycin/penicillin mixture (1%). The supernatant was removed, and the cells were resuspended in DMEM containing 10% fetal bovine serum (FBS) and streptomycin/penicillin mixture (1%) at 37 °C. The cells were dispersed into six-well plates for cultivation.

### 2.7. Prolactin Treatment of Granulosa Cells

GCs were cultured for 72 h in DMEM supplemented with 10% FBS and 1% streptomycin/penicillin mixture. Subsequently, the cells were harvested using trypsin digestion and transferred to sterile 15 mL centrifuge tubes. The cells were washed twice with PBS, collected by centrifugation (1100 rpm for 3 min), and then seeded into 6-well plates at a density of 1 × 10^6^ cells per well. Cells were treated with prPRL at varying concentrations (0, 30, and 300 ng/mL) for a duration of 24 h. Additionally, to simulate the in vivo growth environment of granulosa cells, 1 IU/mL FSH and 0.1 nmol/mL androstenedione were added to all culture groups.

### 2.8. Cell Proliferation Assay

Cell viability of GCs was quantified using a Cell Counting Kit-8 (CCK-8) assay (Beyotime, Shanghai, China). GCs were divided into three experimental groups, each comprising six replicate samples. Cells were seeded in 96-well plates at a density of 3 × 10^3^ cells per well in 100 μL culture medium containing different concentrations of prPRL (0, 30, and 300 ng/mL). After 24 h of cell culture in 96-well plates, 10 μL of CCK-8 reagent was carefully pipetted into each well. The plates were then covered with aluminum foil to maintain darkness and incubated at 37 °C for 3 h to allow the colorimetric reaction to proceed. Absorbance was ultimately measured at 450 nm to assess cell viability as an indicator of proliferation and survival status.

### 2.9. RNA Extraction and Real-Time Quantitative PCR (RT-qPCR)

Total RNA was extracted from GCs and follicular tissue cells using a Qiagen kit (Hilden, Germany) following the manufacturer’s instructions. RNA integrity and concentration were assessed via NanoDrop 2000 (Thermo, Carlsbad, CA, USA), with samples exhibiting A_260_/A_280_ ratios of 1.8–2.0 deemed suitable for downstream applications. RNA was reverse transcribed into cDNA using a reverse transcription kit (Vazyme, Nanjing, China). qPCR amplification was subsequently performed on a qTOWER^3^ system (Analytik Jena, Jena, Germany) under the following cycling protocol: initial denaturation at 95 °C for 40 s, followed by 40 cycles of denaturation at 95 °C for 10 s, annealing at 56 °C for 20 s, and extension at 72 °C for 20 s. In addition, all reactions included non-template controls (NTCs) to rule out potential contamination. Each sample was replicated three times, and relative quantification was conducted utilizing the 2^−ΔΔCt^ method. The expression levels of each target gene were normalized to *GAPDH* expression. The primers utilized for qPCR amplification are enumerated in Supplementary Table S1.

### 2.10. Western Blotting (WB)

Following the manufacturer’s directions, total proteins were obtained from the GCs and follicular tissue using a Whole Protein Extraction Kit (KeyGen Biotech, Nanjing, China). Samples were lysed in RIPA buffer containing phosphatase inhibitors, phenylmethylsulfonyl fluoride (PMSF), and protease inhibitors. The lysates were centrifuged for 5 min at 12,000 rpm, with the supernatant collected as the proteins of the samples. Proteins were separated using 12% SDS–polyacrylamide gels and subsequently transferred to polyvinylidene fluoride membranes (Millipore, Burlington, MA, USA). Membranes were first pretreated with 5% non-fat milk for blocking, followed by incubation with primary antibodies at 4 °C overnight. Subsequently, the membranes were probed with 1:1000 diluted secondary antibodies for 1 h. Protein band quantification was subsequently performed through gray-scale analysis using ImageJ software (v1.8.0). The antibodies used in this study are listed in Supplementary Table S2.

### 2.11. RNA Extraction, Library Preparation, and Sequencing

Total RNA was isolated from GCs (control: CT1, CT2, and CT3; PRL treatment: P1, P2, and P3). RNA integrity and concentration were systematically assessed using an Agilent 2100 Bioanalyzer (Agilent Technologies, Santa Clara, CA, USA). Following the manufacturer’s protocols, cDNA libraries were constructed involving poly(A) selection for mRNA enrichment, fragmentation into sequencing-compatible lengths, and reverse transcription into cDNA. The resulting cDNA fragments underwent end repair, 3′-adenylation, and sequencing adapter ligation. Libraries were then PCR-amplified and quality-controlled, and paired-end sequencing was performed on an Illumina platform. Raw data quality was evaluated using FastQC (v0.11.9) for base quality, GC content, and duplication levels. Adapter trimming and low-quality base removal were conducted with Trimmomatic (v0.39) under default parameters. Cleaned reads were aligned to the Sus scrofa 11.1 reference genome using HISAT2 (v2.2.4). Transcriptome assembly was ultimately performed with StringTie (v2.2.1), completing the bioinformatic pipeline. Gene expression levels were quantified using the fragments per kilobase of transcript per million mapped reads (FPKM) method to normalize for gene length and sequencing depth. To ensure data reliability, sequencing quality metrics, read distribution across genomic regions and chromosomal density were systematically assessed. Additionally, principal component analysis and sample correlation analyses were performed to evaluate the consistency and reproducibility of biological replicates.

### 2.12. Differential Expression and Functional Enrichment Analysis of mRNAs

The transcriptome expression data were processed using DESeq2 software (v1.38.3) to screen for differentially expressed genes and to compare the two groups. |log_2_ Fold Change| ≥ 1 and *p* < 0.05 were used as the screening criteria in the detection process. Genes or transcripts with significant differences in expression levels were grouped into differentially expressed gene (DEG) sets, and intergroup comparisons were made to obtain differentially expressed genes, as well as the statistical significance of the differentially expressed genes. Volcano and heatmaps were used to represent the differences in gene expression levels in the two sets of samples. In addition, we performed gene ontology (GO) annotation of the DEG and Kyoto Encyclopedia of Genes and Genomes (KEGG) enrichment analysis using the KEGG direct homology-based annotation system (KOBAS). The primers used to validate the transcriptome results are listed in Supplementary Table S3.

### 2.13. Statistical Analysis

Statistical analyses were performed using GraphPad Prism 9.0 (GraphPad Software, San Diego, CA, USA) and SPSS 22.0 (IBM, Armonk, NY, USA). Data are expressed as the mean ± standard error of the mean (SEM) from at least three independent experiments. Before conducting the analysis, the data’s normal distribution was assessed through the Shapiro–Wilk test, while Levene’s test was used to ensure equal variances across groups. When comparing more than two groups, a one-way ANOVA was performed, followed by Tukey’s post hoc test to identify specific differences. Results were considered statistically significant if the *p*-value < 0.05.

## 3. Results

### 3.1. Prolactin Concentrations in the Follicular Fluid of Different Follicle Sizes

**Figure 2:**
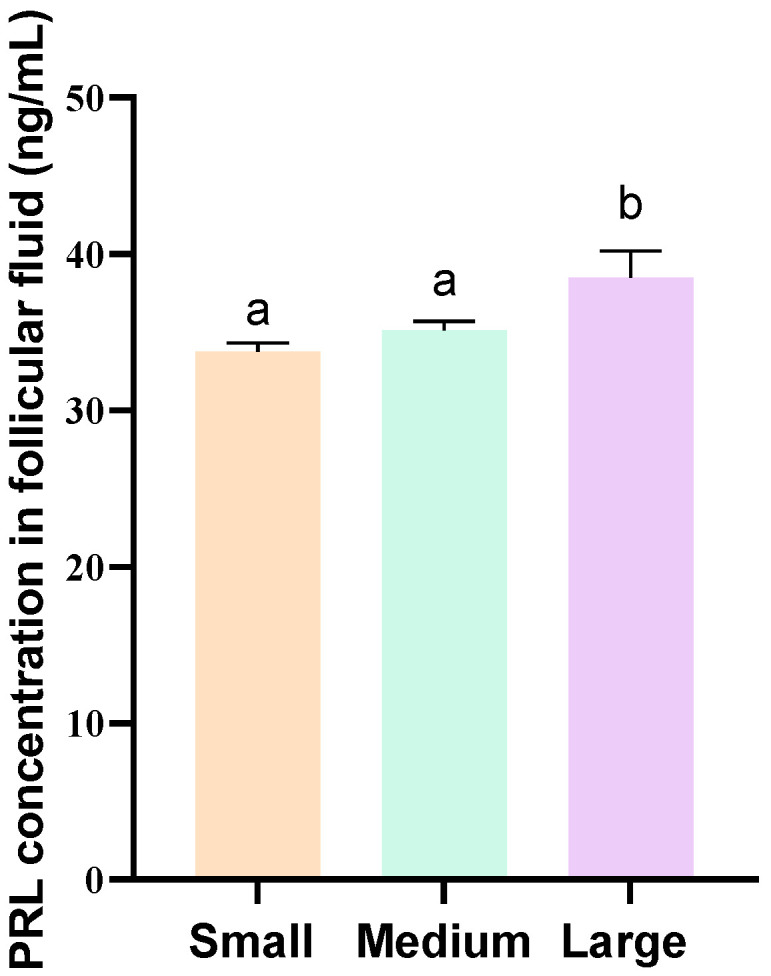
PRL levels in follicular fluids collected from small, medium, and large follicles. Different lowercase letters indicate significant differences (*p* < 0.05); the same letters indicate that the difference is not significant (*p* ≥ 0.05). PRL, prolactin.

The levels of PRL in the follicular fluid of pigs with varying follicle diameters were quantified using ELISA. The results showed that the concentrations of PRL in large follicles were significantly higher than those in medium and small follicles (*p* < 0.05; Figure 2), and the differences in PRL concentrations between small and medium follicles were not significant (*p* ≥ 0.05).

### 3.2. The Expression of Prolactin, Prolactin Receptor, Proteolytic Enzymes, and Key Angiogenesis Factors in Follicular Tissue Cells

**Figure 3:**
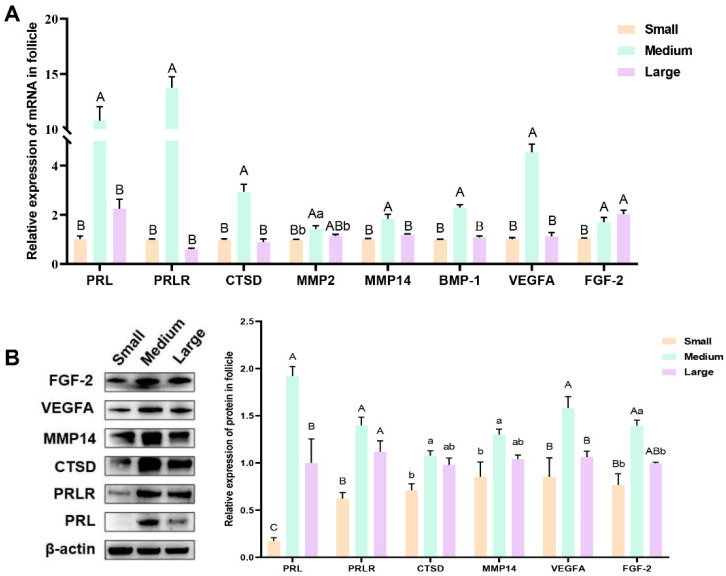
Expression of PRL, PRLR, proteolytic enzymes, and key angiogenic factors in porcine follicular fluid. (**A**) Gene expression in porcine follicles. (**B**) Quantification of protein expression levels using ImageJ (v1.8.0) analysis of Western blotting. Different uppercase letters indicate significant differences (*p* < 0.01); different lowercase letters indicate significant differences (*p* < 0.05); the same letters indicate that the difference is not significant (*p* ≥ 0.05).

The expression of PRL, PRLR, proteolytic enzymes (CTSD, MMP2, MMP14, and BMP-1), and key angiogenesis factors (VEGFA and FGF-2) was analyzed in porcine follicles of different sizes using RT-qPCR and Western blotting. The RT-qPCR results (Figure 3A) revealed that the expression levels of *PRL*, *PRLR*, *CTSD*, *MMP2*, *MMP14*, *BMP-1*, and *VEGFA* were significantly higher in the medium follicles than in the small and large follicles (*p* < 0.05). However, the difference in expression between large and small follicles was not significant (*p* ≥ 0.05). Additionally, *FGF-2* expression was higher in large follicles than in small and medium follicles (*p* < 0.05), although the difference between large and medium follicles was not significant (*p* ≥ 0.05). Western blot analysis (Figure 3B) showed that the expression levels of PRL, PRLR, CTSD, MMP14, VEGFA, and FGF-2 were significantly higher in medium-sized follicles than in small follicles (*p* < 0.05). Additionally, the expression levels of PRL, VEGFA, and FGF-2 were significantly lower in large follicles than in medium follicles (*p* < 0.05). The expression of PRLR, CTSD, MMP14, and FGF-2 showed no significant difference between the large and medium follicles (*p* ≥ 0.05), although a decreasing trend was observed.

### 3.3. Effect of Different Concentrations of Prolactin on Granulosa Cell Proliferation

**Figure 4:**
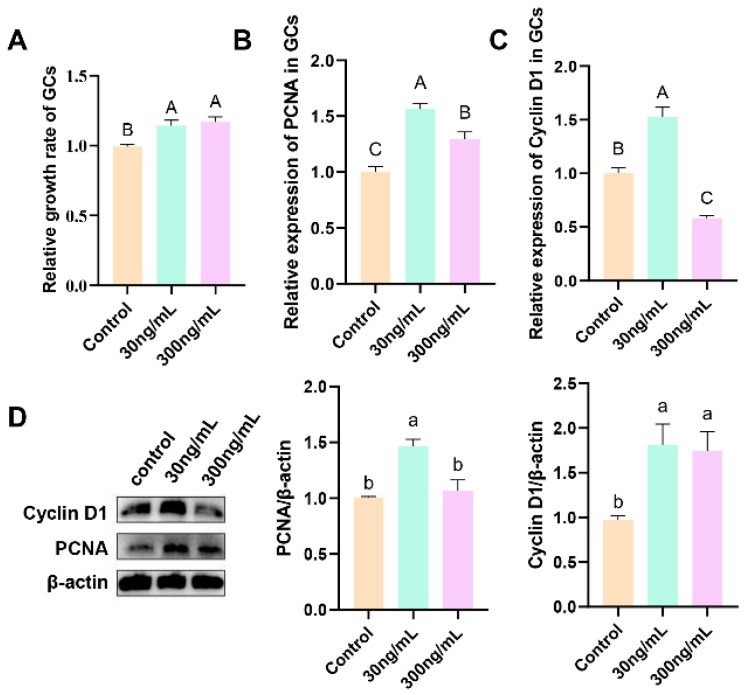
Effect of prPRL on the proliferation of GCs. (**A**) Relative proliferation rate of GCs; (**B**,**C**) Expression of proliferation-related genes of GCs; (**D**) Quantification of protein expression levels using ImageJ (v1.8.0) analysis of Western blotting. Different uppercase letters indicate significant differences (*p* < 0.01); different lowercase letters indicate significant differences (*p* < 0.05); the same letters indicate that the difference is not significant (*p* ≥ 0.05).

Figure 4 shows the cell proliferation rate and relative abundance of PCNA and cyclin D1 mRNA and protein in GCs treated with different concentrations of prPRL (0, 30, and 300 ng/mL) for 24 h. The results show that both 30 and 300 ng/mL prPRL significantly increased the proliferation of GCs (*p* < 0.01; Figure 4A). RT-qPCR analysis revealed that 30 ng/mL prPRL significantly increased the expression of both *PCNA* and *cyclin D1* (*p* < 0.01; Figure 4B,C), and 300 ng/mL prPRL significantly increased PCNA expression (*p* < 0.01; Figure 4B). Western blotting further demonstrated that 30 ng/mL prPRL significantly increased the expression of both PCNA and cyclin D1 (*p* < 0.05), and that 300 ng/mL prPRL significantly increased cyclin D1 expression (*p* < 0.05; Figure 4D).

### 3.4. Effect of Different Concentrations of Prolactin on the Expression of PRLR and Steroidogenesis-Related Genes in Granulosa Cells

**Figure 5:**
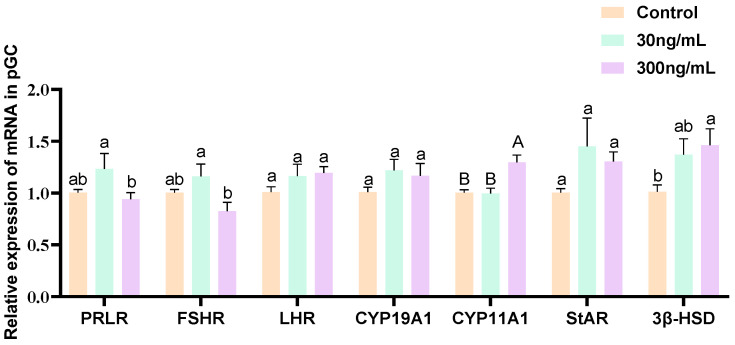
Effect of different concentrations of prPRL on the expression of *PRLR* and steroidogenesis-related genes in GCs. Different uppercase letters indicate significant differences (*p* < 0.01). Different lowercase letters indicate significant differences (*p* < 0.05); the same letters indicate that the difference is not significant (*p* ≥ 0.05).

The relative abundance of *PRLR*, *FSHR*, *LHR*, *CYP19A1*, *CYP11A1*, *STAR*, and *3β-HSD* in GCs treated with different concentrations (0, 30, and 300 ng/mL) of prPRL for 24 h is shown in Figure 5. The results showed that 300 ng/mL prPRL significantly increased the expression of *CYP11A1* in GCs (*p* < 0.05; Figure 5). The results showed that 300 ng/mL prPRL significantly increased the expression of *CYP11A1* and *3β-HSD* in GCs (*p* < 0.05; Figure 5), suggesting that 300 ng/mL prPRL had a facilitating effect on progesterone synthesis in GCs.

### 3.5. Effect of Prolactin on the Expression of Key Angiogenesis Factors in Granulosa Cells

**Figure 6:**
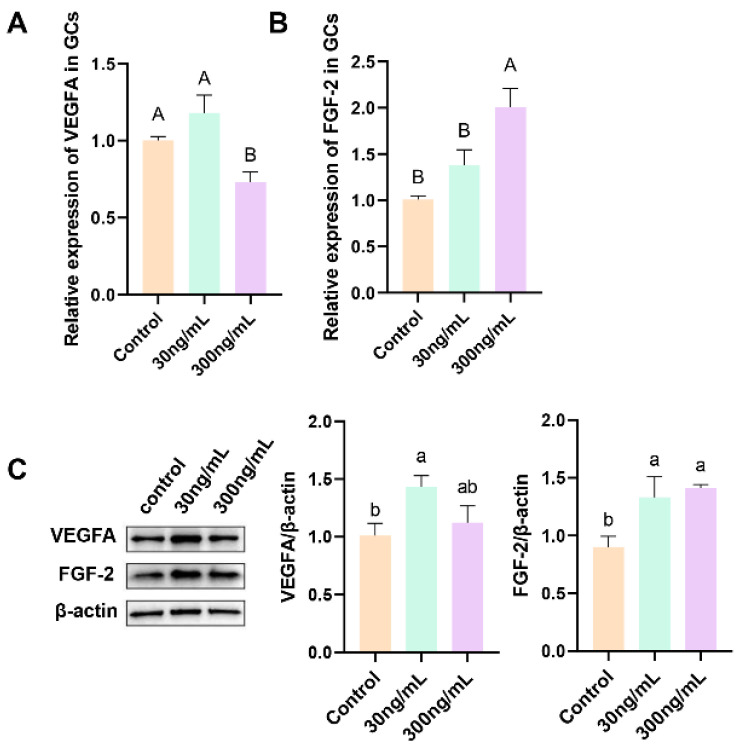
Effect of prPRL on the expression of VEGFA and FGF-2 in GCs. (**A**,**B**) Expression of genes in GCs. (**C**) Quantification of protein expression levels using ImageJ (v1.8.0) analysis of Western blotting. Different uppercase letters indicate significant differences (*p* < 0.01); different lowercase letters indicate significant differences (*p* < 0.05); the same letters indicate that the difference is not significant (*p* ≥ 0.05).

Figure 6 shows the relative abundance of VEGFA and FGF-2 mRNAs and proteins in GCs treated with different concentrations (0, 30, and 300 ng/mL) of prPRL for 24 h. RT-qPCR results showed that 300 ng/mL prPRL enhanced the expression of *FGF-2* (*p* < 0.01; Figure 6B). Western blot analysis showed that 30 ng/mL prPRL enhanced the expression of VEGFA and FGF-2 (*p* < 0.05; Figure 6C), and 300 ng/mL prPRL enhanced the expression of FGF-2 (*p* < 0.05; Figure 6C).

### 3.6. Differentially Expressed Genes Analysis

Six cDNA libraries were constructed from the GCs. Six transcriptome libraries were quality-controlled to remove junctions and low-quality reads, and each library retained an average of approximately 42.42 million clean reads for subsequent bioinformatic analysis. The fundamental details of the sequencing data are presented in Table 1.

**Figure 7:**
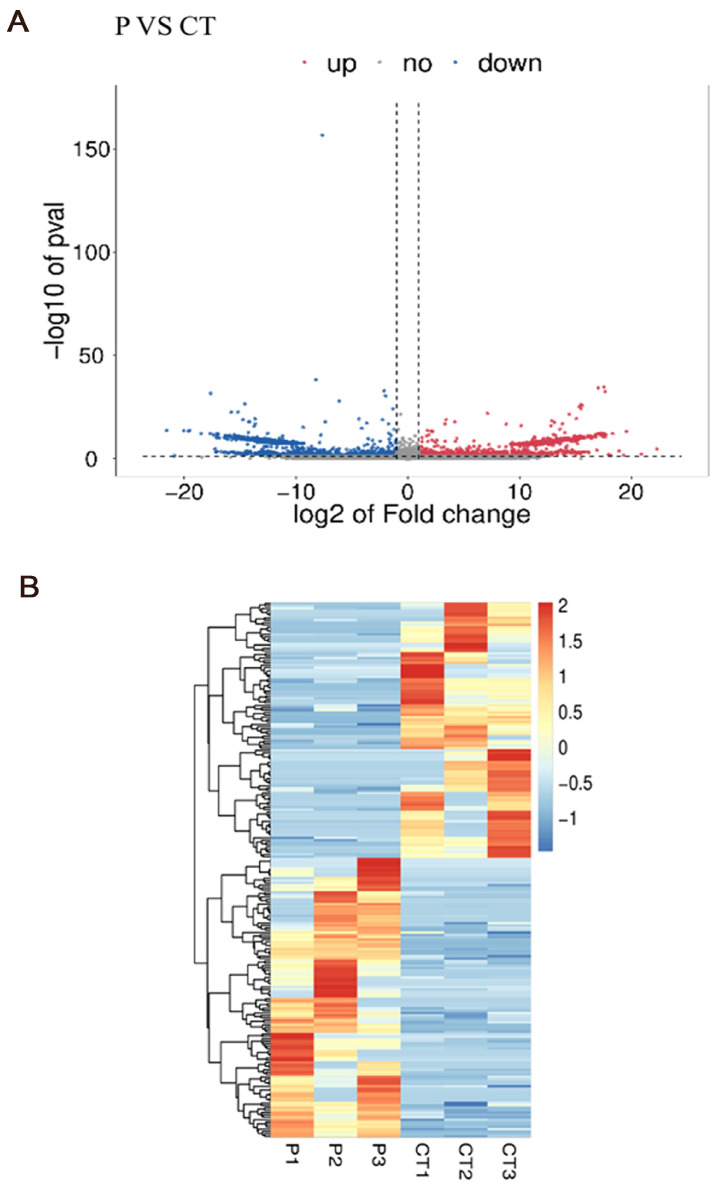
Analysis of the RNA-Seq. (**A**) Volcano map of differentially expressed genes. (**B**) Differential gene expression clustering heatmap. CT: control group (0 ng/mL prPRL); P: GCs treated with 30 ng/mL prPRL.

In total, 21,682 genes were obtained after comparing the sequencing results to the porcine RefSeq database, and the genes were obtained and differentially analyzed using the Ballgown package (v2.22.0). The |log_2_ fold change| > 1 and *p* < 0.05 were used as the parameters to screen for DEGs. The results are shown in Figure 7. A total of 226 DEGs were obtained in the granulosa cell group treated with prPRL compared to the control group, and 118 and 108 genes with significantly upregulated and down-regulated expression, respectively, were detected. Statistical analysis of the expression of differentially expressed genes in the two groups was performed using volcano plots (Figure 7A) and cluster analysis heatmaps (Figure 7B).

### 3.7. Gene Ontology and Kyoto Encyclopedia of Genes and Genomes Pathway Enrichment Analysis

**Figure 8:**
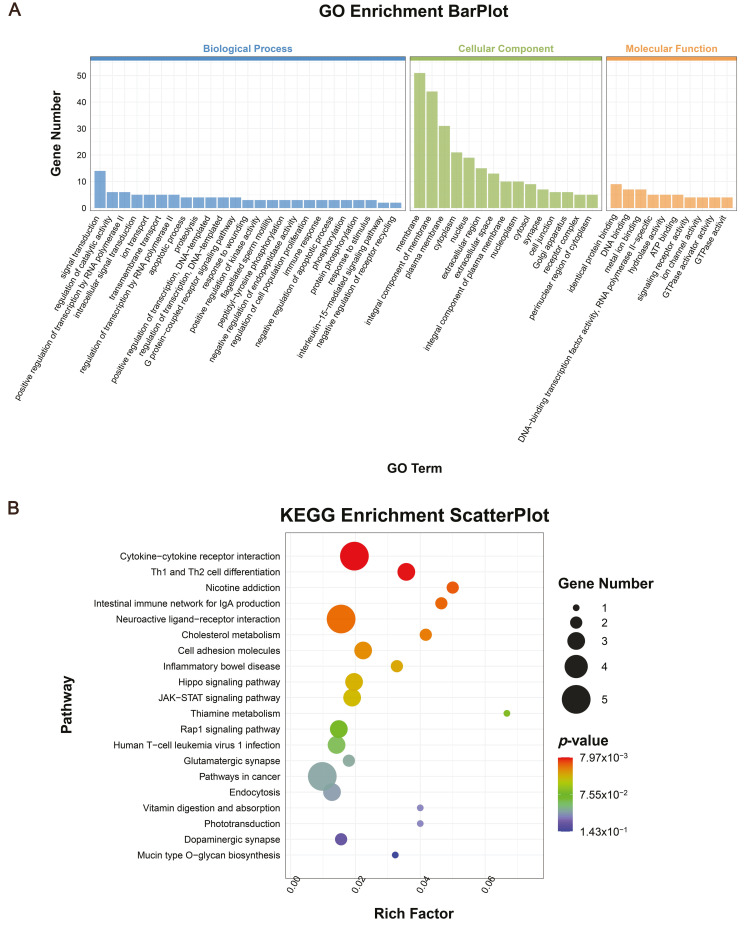
Analysis of the RNA-seq. (**A**) GO functional enrichment analysis of differentially expressed genes; (**B**) Differential gene expression of KEGG-enriched top 20 pathways.

GO functional enrichment analysis was conducted on the DEGs, and the results are presented in Figure 8A. Differentially expressed genes were enriched in cellular components such as the cell membrane, integral membrane components, plasma membrane, cytoplasm, nuclear receptor complex, and various organelles. In terms of biological processes, these genes are involved in signal transduction, cell proliferation, immune responses, catalytic activity regulation, RNA polymerase II transcription, transmembrane transport, and transcriptional regulation. Molecular functions were predominantly enriched for protein binding, GTPase activity, DNA binding, cation binding, hydrolase activity, signal receptor activity, and ATP binding. These findings suggest that PRL may influence the biological processes of GCs, particularly through its effects on cell proliferation and the immune response.

Significantly enriched pathways for DEGs were analyzed using a hypergeometric test. The top 20 pathways (TOP20) with the lowest Q-values for KEGG pathway enrichment significance are shown in Figure 8B. Among these pathways, the DEGs in GCs were primarily enriched in cytokine receptor interactions, cholesterol metabolism, and cell adhesion, and the Hippo, Rap1, and JAK/STAT signaling pathways. These pathways are closely associated with the regulation of cell growth, angiogenesis, and follicular development.

To identify PRL-affected genes, we screened DEGs with FPKM > 0.5, *p* < 0.05, and log_2_ fold change > 1. These genes were further enriched for GO functions and KEGG pathways based on their expression profiles in the two groups. Significantly differentially expressed genes with clear functional descriptions were selected as candidate genes for integrative analysis. *PRKACB*, *DNAJC13*, *PFKFB2*, *TAGLN*, *PCNA*, *MAPK3*, *PPP2R2B*, *HSD11B1*, *DDX54*, *HSD17B4*, and *FDFT1* were associated with cell proliferation and steroid synthesis (Table 2). *DKK3*, *MALT1*, *IL15*, *TNFRSF9*, and *MAPK6* were linked to the immune response (Table 2). In addition, *TWSG1*, *MRTFA*, *SCG2*, and *ANGPTL7* were associated with angiogenesis (Table 2).

### 3.8. Real-Time Quantitative PCR Confirmation

**Figure 9:**
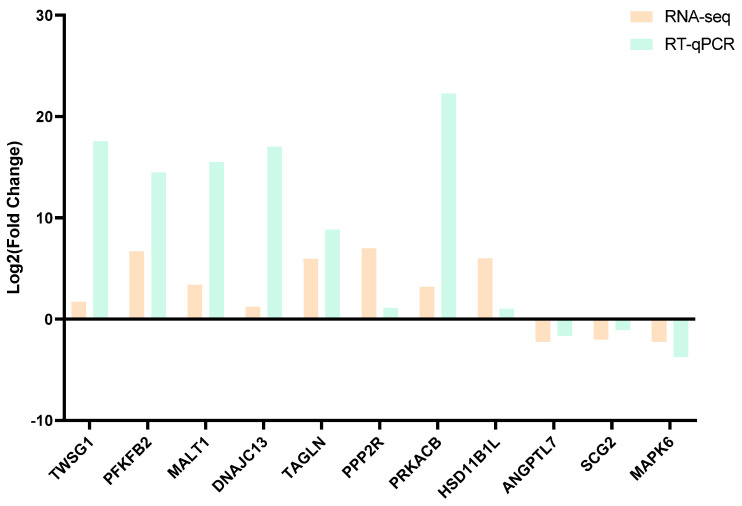
RT-qPCR validation of differentially expressed genes.

To validate the differential expression of these genes, 11 genes were selected for RT-qPCR: *TWSG1*, *ANGPTL7*, *HSD11B1L*, *SCG2*, *MAPK6*, *MALT1*, *DNAJC13*, *TAGLN*, *PPP2R2B*, *PFKFB2*, and *PRKACB* (Figure 9). The results of the RT-qPCR analysis are illustrated in Figure 9, confirming the reliability of the transcriptome sequencing data.

## 4. Discussion

In this research, we integrated in vivo and in vitro experimental methods to investigate the expression patterns of PRL, proteolytic enzymes, angiogenesis-related genes, and other related genes during various stages of follicular development. Furthermore, we revealed potential molecular signaling pathways through which PRL regulates these processes. The results showed that PRL not only promotes the proliferation and angiogenesis of GCs but may also achieve its regulatory effects through the Hippo, JAK/STAT and Rap1 signaling pathways.

Previous research has shown that PRL is present in porcine follicular fluid and the *PRL* gene is expressed in porcine ovaries [22,23]. GCs synthesize and secrete PRL [24]. These autocrine and paracrine roles may significantly affect follicular physiology. In our study, ELISA was used to measure PRL concentrations in follicular fluid from follicles of different sizes, with PRL levels ranging from 32.3 to 42.7 ng/mL. These concentrations increased with follicle development, aligning with prior research findings [11,25]. Follicular development is associated with granulosa cell proliferation. Previous studies have demonstrated that low concentrations of PRL (4 ng/mL or 50 ng/mL) significantly enhance sheep granulosa cell viability. In contrast, high concentrations of PRL (500 ng/mL) have been shown to induce granulosa cell apoptosis by triggering oxidative stress and activating autophagy pathways [26]. These findings highlight the dose-dependent nature of PRL’s effects on GCs. Our findings are largely consistent with the above observations. Treatment with prPRL at 30 ng/mL and 300 ng/mL significantly enhanced granulosa cell viability and upregulated the expression of proliferation-related genes, including PCNA and cyclin D1. Notably, our results showed that 300 ng/mL failed to inhibit granulosa cell viability, which may be due to the fact that our dose has not reached a high dose, but this needs further study on its effect on granulosa cell apoptosis. These results indicate that PRL plays a crucial regulatory role in the follicle, supports granulosa cell function and follicle growth, and reflects the physiological state and health of the ovary. Moreover, the effect of PRL on granulosa cell proliferation depends on the dose of PRL.

PRL plays a key regulatory role in the process of angiogenesis, especially in promoting endothelial cell proliferation and angiogenesis [27,28]. Follicular growth is highly dependent on adequate angiogenesis, which ensures continuous delivery of oxygen and nutrients. Previous research has identified PRL in follicular fluid as a major mitogenic factor for follicular endothelial cells, highlighting its role in driving endothelial proliferation during angiogenesis [29]. The full-length form of PRL (23 kDa) promotes angiogenesis, whereas the protein-hydrolyzed isoform of PRL, Vi, exhibits anti-angiogenic properties [30]. In this study, we examined the expression patterns of PRL, its receptor (PRLR), key proteolytic enzymes (CTSD, MMP2, MMP14, and BMP-1), and angiogenic factors (VEGFA and FGF2) in porcine follicles at different developmental stages. Our results revealed a dynamic expression pattern characterized by an initial increase followed by a decline during follicular maturation, consistent across PRL, PRLR, proteases, and angiogenic markers. Combined with ELISA data showing fluctuating PRL concentrations in follicular fluid, we propose that during early follicular development, elevated PRLR expression and increased local PRL synthesis may activate angiogenic pathways, facilitating neovascularization within the follicular microenvironment. As follicles mature, PRL appears to shift from intra-tissue accumulation to release into follicular fluid, suggesting a transition from autocrine to paracrine regulatory mechanisms. Interestingly, the expression trends of VEGFA and FGF2 mirrored those of PRL-cleaving enzymes, implying a potential coordination between the angiogenic and proteolytic systems. This balance may ensure that angiogenesis is precisely regulated, with vascular expansion promoted when needed and restrained through Vi generation to prevent excessive vessel growth. Such a mechanism likely contributes to the establishment of a functional vascular network that supports follicular development while maintaining homeostasis.

Follicular development is precisely regulated by a complex network of endocrine and paracrine signals secreted by GCs, among which the steroid hormones estradiol (E2) and progesterone (P4) play pivotal roles in maintaining follicular growth and function [31,32]. E2 promotes GC differentiation and follicle antrum formation, and enhances granulosa cell sensitivity to FSH [33]; while P4 is crucial for ovulation and corpus luteum formation [34]. Studies have shown that PRL plays an important regulatory role in steroidogenesis by modulating the expression of key steroidogenic enzymes [35,36]. Among these, cytochrome P450 side-chain cleavage enzyme (CYP11A1) and 3β-hydroxysteroid dehydrogenase (3β-HSD) are critical rate-limiting enzymes: CYP11A1 converts cholesterol into pregnenolone, and 3β-HSD further catalyzes its conversion into P4 [37,38]. Experimental evidence has shown that PRL can significantly upregulate the expression of these enzymes, thereby enhancing steroid hormone biosynthesis. He et al. [36] reported that treatment of ovine gastric cancer cells with 50 or 500 ng/mL PRL significantly upregulated CYP11A1 expression and enhanced P4 production. Similarly, Hu et al. [2] found in a poultry model that low-dose PRL significantly increased the expression of 3β-HSD and CYP11A1 in GCs and promoted the synthesis of E2 and P4, while high-dose PRL weakened this effect, suggesting a dose-dependent regulatory mechanism. Consistent with these findings, our study revealed that 300 ng/mL PRL significantly upregulated 3β-HSD and CYP11A1 expression in GCs, indicating that PRL may enhance progesterone production through activation of these steroidogenic enzymes. Furthermore, the differential response to PRL dosage observed across species suggests that PRL’s effects on steroidogenesis may be influenced by species-specific sensitivity, receptor expression levels, and follicular stage. In addition to granulosa cells, PRL has also been shown to stimulate P4 production in theca and luteal cells [39,40], indicating its broad regulatory role in ovarian steroidogenesis. It is also notable that PRL-mediated regulation may be further modulated by feedback mechanisms involving FSH, luteinizing hormone, and local growth factors, which warrants further investigation.

Ovarian and follicular function relies on sustained angiogenesis to ensure sufficient oxygen and nutrient supply during follicular development. VEGFA, a key angiogenic factor, plays a central role in this process by acting on granulosa and theca cells and promoting neovascularization within the follicle [41,42]. Together with FGF2, another potent angiogenic factor, VEGFA stimulates endothelial cell proliferation and migration, supporting vascular formation and stability [43,44]. In this study, 30 ng/mL prPRL significantly upregulated VEGFA and FGF2 expression in GCs. However, at 300 ng/mL, PRL further increased FGF2 but had no significant effect on VEGFA. This partially differs from Basini et al. [11], who reported increased VEGFA only at high PRL doses. The discrepancy may relate to follicle size, as our GCs were derived from medium follicles, while theirs were from large follicles. Our in vivo results also showed higher VEGFA expression in medium follicles, suggesting stage-specific responsiveness to PRL. Moreover, VEGFA and FGF2 have been shown to synergistically promote steroidogenesis and GC proliferation while inhibiting apoptosis [45,46]. These findings support the hypothesis that PRL may enhance angiogenesis and steroid production by upregulating VEGFA and FGF2 in a dose- and stage-dependent manner.

To elucidate the molecular mechanisms by which PRL regulates follicular development, we performed RNA sequencing on GCs treated with prPRL. A total of 226 DEGs were identified, including 118 upregulated and 108 downregulated genes. Among them, proliferation-related genes such as *PCNA* and *MAPK3* were significantly upregulated, as were angiogenesis-related genes *TWSG1* and *MRTFA*. Conversely, immune-related genes such as *IL15* were significantly downregulated. In addition, RT-qPCR verification showed that the sequencing results were reliable. GO enrichment analysis indicated that these DEGs were primarily involved in signal transduction, cell proliferation, immune regulation, and transcriptional control. KEGG pathway analysis revealed significant enrichment in the Hippo, JAK/STAT, Rap1, and Th1/Th2 cell differentiation signaling pathways. Prior studies have shown that PRL activates downstream cascades such as PI3K/AKT, MAPK, and JAK/STAT via PRLR binding, thereby regulating cellular proliferation, differentiation, and apoptosis [47]. Although these mechanisms have been described in other cells, evidence in porcine GCs remains limited. In this study, the enrichment of the Hippo and JAK/STAT pathways, along with the upregulation of key genes like *PCNA*, *MAPK3*, *TWSG1*, and *MRTFA*, suggests that PRL may promote follicular development by enhancing granulosa cell proliferation and angiogenesis through these pathways. In addition, Rap1 signaling, known for linking extracellular signals to intracellular responses, can activate MAPK and PI3K/AKT cascades to regulate cell adhesion, migration, and proliferation [48]. PRL-induced upregulation of MAPK3, a key Rap1 downstream effector, further supports the involvement of the Rap1/MAPK axis in PRL-mediated granulosa cell function. Regarding immune modulation, PRL has been shown to influence T and B lymphocyte activity, although its effects on immune responses are complex and dose-dependent [49]. Matalka et al. [50] found that 15–30 ng/mL of PRL can promote the secretion of immune factors such as IL-12 by helper T cells (Th1), but when the concentration exceeds 100 ng/mL, the effect is significantly weakened. Similarly, Ellah et al. [51] found that PRL inducers can suppress pro-inflammatory cytokines such as IL-6 and restore immune homeostasis. In our study, 30 ng/mL PRL significantly reduced IL15 expression in GCs, suggesting that PRL may suppress certain immune-related factors in a cell-type and context-dependent manner. Moreover, the balance between Th1/Th2 cell subsets plays a crucial role in maintaining the ovarian immune microenvironment and modulating angiogenesis [52]. PRL has been proposed to influence this balance, thereby linking immune regulation with vascular remodeling [53]. In summary, our findings suggest that PRL may regulate granulosa cell proliferation, angiogenesis, steroidogenesis, and immune responses through multiple pathways, including Hippo, JAK/STAT, and Rap1, ultimately contributing to the orchestration of follicular development.

## 5. Conclusions

In conclusion, PRL may regulate follicular development by modulating the expression of the protein hydrolases CTSD, MMP2, MMP14, and BMP-1, and the key angiogenic factors VEGFA and FGF-2. PrPRL at 30 and 300 ng/mL promoted granulosa cell proliferation and expression of the key angiogenic factors VEGFA and FGF-2. PRL regulates granulosa cell proliferation and angiogenesis to regulate follicular development via the JAK/STAT, Hippo, and Rap1 signaling pathways.

## Figures and Tables

**Table 1:** Detailed information on RNA sequencing.

Sample	Raw Reads	Raw Bases	Clean Reads	Clean Bases	Q20	Q30	GC (%)
CT1	45,683,498	6.85	44,556,134	6.68	99.98	96.33	51
CT2	46,050,912	6.91	44,719,212	6.71	99.97	96.28	51
CT3	45,777,432	6.87	44,505,288	6.68	99.98	96.51	51
P1	45,994,936	6.90	44,730,614	6.72	99.97	96.39	51
P2	38,084,340	5.71	37,043,024	5.56	99.98	96.68	51
P3	40,008,974	6.00	38,965,208	5.84	99.98	96.67	51.5

**Table 2:** Differentially expressed genes induced by recombinant porcine prolactin.

Gene Function	Genes	Description	Log_2_FC
Cell proliferation-related differential genes	*PRKACB*	Protein kinase cAMP-activated catalytic subunit beta	22.28
	*DNAJC13*	DNAJ heat shock protein family (Hsp40) member C13	17.03
	*PFKFB2*	6-Phosphofructo-2-kinase/fructose-2,6-biphosphatase 2	14.47
	*TAGLN*	Transgelin	8.83
	*PCNA*	Proliferating cell nuclear antigen	3.34
	*MAPK3*	Mitogen-activated protein kinase 3	1.32
	*PPP2R2B*	Protein phosphatase 2 regulatory subunit beta	1.12
	*HSD11B1*	Hydroxysteroid 11-beta dehydrogenase 1	1.03
	*DDX54*	DEAD-box helicase 5	−16.44
	*HSD17B4*	Hydroxysteroid 17-beta dehydrogenase 4	−13.06
	*FDFT1*	Farnesyl-diphosphate farnesyltransferase 1	−12.59
Immune response-related differential genes	*DKK3*	Dickkopf wnt signaling pathway inhibitor 3	19.57
	*MALT1*	MALT1 paracaspase	15.51
	*IL15*	Interleukin 15	−9.54
	*TNFRSF9*	TNF receptor superfamily member 9	−8.72
	*MAPK6*	Mitogen-activated protein kinase 6	−3.75
Angiogenesis-related differential genes	*TWSG1*	Twisted gastrulation BMP signaling modulator 1	17.56
	*MRTFA*	Myocardin-related transcription factor A	17.65
	*SCG2*	Secretogranin II	−1.07
	*ANGPTL7*	Angiopoietin-like 7	−1.64

## Data Availability

The raw data from sequencing can be found below: NCBI SRA (accession: PRJNA1225948).

## Supplementary Materials

The following supporting information can be downloaded at: https://www.mdpi.com/article/10.3390/genes16070774/s1. Table S1: Primer sequences of the porcine GCs and follicles for qPCR; Table S2: Information on the antibodies used in this study; Table S3: Primer sequences for validation of transcriptome sequencing results.
