# Supplementary material for: Transcriptomic Analysis Reveals the Molecular Mechanisms of Prolactin in Regulating Porcine Follicular Development
Source: Genes (Basel). 2025 Jun 30;16(7):774. doi: 10.3390/genes16070774 (PMC12296069; doi:10.3390/genes16070774)
Supplement: Supplementary file 1 [file genes-16-00774-s001.zip › Supplementary Material.pdf]

Supplementary Material

Supplementary Tables

Table S1 Primer sequences of the porcine GCs and follicles for qPCR

**Target gene**	**Accession number**	**Primer sequence (5′ to 3′)**	**Length(bp)**
GAPDH-F	NM_001206359.1	CCCCAACGTGTCGGTTGT	91
GAPDH-R		CTCGGACGCCTGCTTCAC	
PRL-F	NM_213926.1	AGCAAGCCCAACAGATCCAT	100
PRL-R		CTCACTTCCGTGACCAGGTG	
PRLR-F	NM_001001868	ACAGTCACCTCCGGGAAAAC	179
PRLR-R		TAGGGCCGCCAGTTTTGTAG	
CTSD-F	NM_001037721.1	TTCACCGTCGTCTTCGACAC	103
CTSD-R		CGCTGTTGTACTTGTGGTGG	
MMP2-F	NM_214192.3	AGGATGGCAAGTACGGCTTC	114
MMP2-R		AGCTGTTGTAGGATGTGCCC	
MMP14-F	XM_021098303.1	GCAAACAGGCGCTTTCAAGA	90
MMP14-R		AGCCTTTGTCTTCGGTAGGC	
BMP1-F	XM_021072336.1	ACAAGGACGAGTGCTCCAAG	155
BMP1-R		GGTGGATGTCACCTTGTGGT	
VEGFA-F	NM_214084.2	CTCACCAAGGCCAGCACATA	127
VEGFA-R		AAATGCTTTCTCCGCTCCGA	
FGF-2-F	NM_001405514.1	TATCTTCTCCAGGCTCCGACA	187
FGF-2-R		AATTGGGGTGCAGGTACCAA	
LHR-F	XM_021085886.1	AAGCACAGCAAGGAGACCAA	231
LHR-R		AAGAGGACAGTCACGTTTCC	
FSHR-F	XM_021085884.1	GAATTGAAAAGGCCAACAAC	214
FSHR-R		CTTTCAAAACTTAGTCCCACG	
CYP19A1-F	NM_214429.1	TCCGCAATGACTTGGGCTAC	103
CYP19A1-R		GCCTTTTCGTCCAGTGGGAT	
CYP11A1-F	XM_021098320.1	GGGCAACCCATTTCCTACCA	95
CYP11A1-R		CGAGCACTGGTGGTACAGAC	
STAR-F	NM_213755.3	CGTTTAAGCTGTGTGCTGGG	132
STAR-R		TCCATGACCCTGAGGTTGGA	
3β-HSD-F	XM_021088745.1	CCTTCAATCGCCACTTCG	157
3β-HSD-R		TCCTTGTGCTGCTTCACCA	
PCNA-F	NM_001291925.1	GCAGAGCATGGACTCGTCTC	120
PCNA-R		TTGGACATGCTGGTGAGGTT	
cyclin D1-F	XM_021082686.1	CTGGTGCCAACTGGTGTTTG	176
cyclin D1-R		CGTACTGGCCTTACGAGCAT	

Table S2. Information on the antibodies used in this study

Article Number	Antibodies	Dilution Ratio	Place of Origin
M1210-2	anti-β-actin	1:10000	Huaan, Hangzhou, China
ER1803-72	anti-PRL	1:1000	Huaan, Hangzhou, China
ER1915-43	anti-PRLR	1:1000	Huaan, Hangzhou, China
ET1608-49	anti-CTSD	1:1000	Huaan, Hangzhou, China
ET1606-48	anti-MMP-14	1:1000	Huaan, Hangzhou, China
R26073	anti-VEGFA	1:1000	Zenbioscience, Chengdu, China
R22504	anti-FGF-2	1:1000	Zenbioscience, Chengdu, China
60097-1-Ig	anti-PCNA	1:5000	Proteintech, Wuhan, China
ET1601-31	anti-Cyclin D1	1:1000	Huaan, Hangzhou, China

Table S3. Primer sequences for validation of transcriptome sequencing results

Target gene	Accession number	Primer sequence (5′ to 3′)	Length(bp)
ANGPTL7-F	NM_001142828.2	CGCCCTCAGCTATCACAACA	142
ANGPTL7-R		GTAGTACACGCCGTTGAGGT	
HSD11B1L-F	XM_013994748.2	GAGAGGGGAAGGGTTTGTGG	194
HSD11B1L-R		CAGATGCAGCCCACACTTTG	
SCG2-F	NM_001012299.2	AACCGGCAAATGGCCTATGA	125
SCG2-R		TGACTGGGAACTCGCTTCAC	
MAPK6-F	XM_001925291.5	CATCCCAAGGCAAGCATGAA	153
MAPK6-R		GCTGGCCACGTTTTATTTGTCC	
TWSG1-F	XM_003127831.4	GCTGGGGGTTCTAACGGAAA	265
TWSG1-R		TTGCCATGAGGGGCACATAC	
MALT1-F	XM_005668244.3	ATTGCTGTGGCTCTTGCGTAGG	266
MALT1-R		TTAACCAACTGAGGGAGGCTAGGG	
DNAJC13-F	XM_021069457.1	GCAGTAACGCAGGATGGTCTCTTC	281
DNAJC13-R		ATGAGGGCACACAGCATATCAACAG	
TAGLN-F	XM_005667371.3	GAAGAAAGCCCAGGAGCATAAGAGG	138
TAGLN-R		ATCTGCCGAGGTCGTCCGTAG	
PPP2R-F	NM_214025.2	CACAACTACCTCCGCAGCAAGC	85
PPP2R-R		CTGACCCATTCCACACGCACTC	
PFKFB2-F	XM_021063329.1	CACACGCTACCTCAACTGGATTGG	73
PFKFB2-R		GACCGCTTCACGCCGATACAC	
PRKACB-F	XM_021096368.1	TTGCTGGTCAGTTGCTTGAGGATAC	267
PRKACB-R		ACAGGCTGGTCACCGAGTTACTC	
